# Benchmarking Undedicated Cloud Computing Providers for Analysis of Genomic Datasets

**DOI:** 10.1371/journal.pone.0108490

**Published:** 2014-09-23

**Authors:** Seyhan Yazar, George E. C. Gooden, David A. Mackey, Alex W. Hewitt

**Affiliations:** 1 Centre for Ophthalmology and Visual Science, University of Western Australia, Lions Eye Institute, Perth, Western Australia, Australia; 2 School of Medicine, Menzies Research Institute Tasmania, University of Tasmania, Hobart, Tasmania, Australia; 3 Centre for Eye Research Australia, University of Melbourne, Department of Ophthalmology, Royal Victorian Eye and Ear Hospital, Melbourne, Victoria, Australia; Saint Louis University, United States of America

## Abstract

A major bottleneck in biological discovery is now emerging at the computational level. Cloud computing offers a dynamic means whereby small and medium-sized laboratories can rapidly adjust their computational capacity. We benchmarked two established cloud computing services, Amazon Web Services Elastic MapReduce (EMR) on Amazon EC2 instances and Google Compute Engine (GCE), using publicly available genomic datasets (*E.coli* CC102 strain and a Han Chinese male genome) and a standard bioinformatic pipeline on a Hadoop-based platform. Wall-clock time for complete assembly differed by 52.9% (95% CI: 27.5–78.2) for *E.coli* and 53.5% (95% CI: 34.4–72.6) for human genome, with GCE being more efficient than EMR. The cost of running this experiment on EMR and GCE differed significantly, with the costs on EMR being 257.3% (95% CI: 211.5–303.1) and 173.9% (95% CI: 134.6–213.1) more expensive for *E.coli* and human assemblies respectively. Thus, GCE was found to outperform EMR both in terms of cost and wall-clock time. Our findings confirm that cloud computing is an efficient and potentially cost-effective alternative for analysis of large genomic datasets. In addition to releasing our cost-effectiveness comparison, we present available ready-to-use scripts for establishing Hadoop instances with Ganglia monitoring on EC2 or GCE.

## Introduction

Through the application of high-throughput sequencing, there has been a dramatic increase in the availability of large-scale genomic datasets [1]. With reducing sequencing costs, small and medium-sized laboratories can now easily amass many gigabytes of data. Given this dramatic increase in the volume of data generated, researchers are being forced to seek efficient and cost-effective measures for computational analysis [2]. Cloud computing offers a dynamic means whereby small and medium-sized laboratories can rapidly adjust their computational capacity, without concern about its physical structure or ongoing maintenance [3]–[6]. However, transitioning to a cloud environment presents with unique strategic decisions [7], and although a number of general benchmarking results are available (http://serverbear.com/benchmarks/cloud; https://cloudharmony.com/; Accessed 2014 Aug 7), there has been a paucity of comparisons of cloud computing services specifically for genomic research.

**Figure 1:**
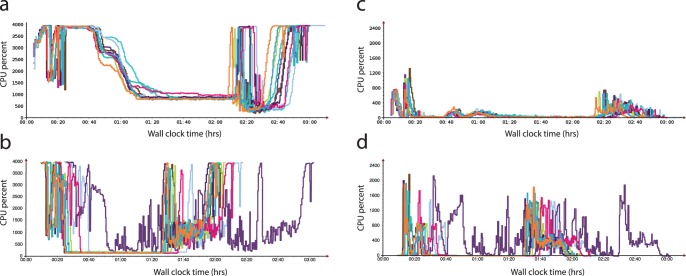
Comparison of undedicated cloud computing performances. The panel includes results of Amazon Web Services Elastic MapReduce (EMR) on Amazon EC2 instances (panels a & c) versus Google Compute Engine (GCE) (panels b & d) for human genome alignment and variant calling. In this 40 node cluster the total CPU percent for CPU idle (a and b) and waiting for disk input/output (c and d) is displayed. Note the greater consistency in performance of Crossbow, though generally longer wall clock times for complete analysis, on EMR compared to GCE.

**Figure 2:**
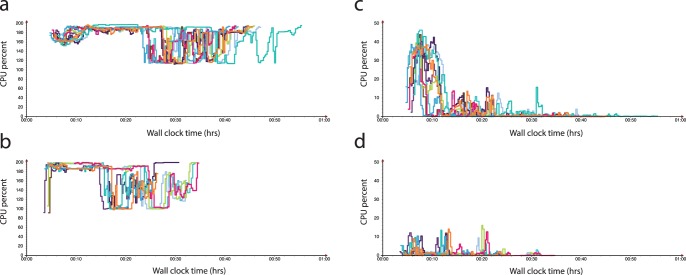
Comparison of undedicated cloud performance of Amazon Web Services Elastic MapReduce (EMR) on Amazon EC2 instances (panels a & c) versus Google Compute Engine (GCE) (panels b & d) for *E.coli* genome alignment and variant calling. In this two node cluster the total CPU percent for CPU idle (a and b) and waiting for disk input/output (c and d) is displayed. Note the shorter wall clock times for complete analysis on GCE compared to EMR.

We undertook a performance comparison on two established cloud computing services: Amazon Web Services EMR on Amazon EC2 instances and GCE. Paired-end sequence reads of publicly available genomic datasets (*Escherichia coli* CC102 strain and a Han Chinese male genome) were analysed using Crossbow, a genetic annotation tool, on Hadoop-based platforms with equivalent system specifications [8]–[10]. A standard analytical pipeline was run simultaneously on both platforms multiple times (Figure 1 and 2). The performance metrics of both platforms were recorded using Ganglia, an open-source high performance computing monitoring system [11].

## Results

Wall-clock time for complete mapping and SNP calling differed by 52.9% (95% CI: 27.5–78.2) and 53.5% (95% CI: 34.4–72.6) for *E.coli* and human genome alignment and variant calling, respectively, with GCE being more efficient than EMR. Table 1 displays the key metrics for data analysis using both services. The proportion of central processing unit (CPU) usage by Crossbow differed between platforms when aligning and SNP calling each genome, with GCE having better utilisation as the genome size increased. There was considerably more free memory on GCE for the smaller *E.coli* dataset and on EMR for larger human genome runs. The CPU idle percentage, the percentage of time where the CPU was idle without waiting for disk input/output (I/O), was greater on EMR for the human genome while CPU waiting for I/O (WIO) was considerably lower on the same platform. The CPU idle and CPU WIO percentages were both significantly higher on EMR for the *E.coli* genome. The cost of running this Crossbow pipeline on EMR and GCE also differed significantly (p<0.001), with the costs on EMR being 257.3% (95% CI: 211.5–303.1) and 173.9% (95% CI: 134.6–213.1) more expensive than GCE for *E.coli* and human assemblies, respectively. For ∼36x coverage of a human genome, at a current sequencing cost of ∼US$1000, the median cost for computation on GCE was US$29.81 (range: US$28.86 to US$45.99), whilst on EMR with a fixed hourly rate it was US$69.60 (range: US$69.60 to US$92.80).

**Table 1:** Comparison of performance metrics for genomic alignment and SNP calling.

	*E.coli* Genome			Human Genome		
Metric	EMR (n = 10)	GCE (n = 10)	p-value*	EMR (n = 10)	GCE (n = 10)	p-value*
Wall clock time (mean)	0∶46∶30	0∶31∶50	<0.001	2∶58∶24	2∶14∶12	<0.001
Pre-processing short readstime (mean)	0∶14∶37	0∶12∶46	0.109	0∶07∶29	0∶06∶23	0.116
Alignment with Bowtietime (mean)	0∶07∶04	0∶05∶03	<0.001	1∶51∶06	1∶15∶07	0.003
Calling SNPs with SOAPsnptime (mean)	0∶05∶05	0∶02∶51	<0.001	0∶35∶31	0∶29∶31	0.033
Post-processing time (mean)	0∶04∶51	0∶00∶57	<0.001	0∶01∶23	0∶01∶03	<0.001
CPU user (mean %)	17.44±1.30	22.31±3.14	<0.001	43.80±1.87	58.05±6.20	<0.001
CPU idle (mean %)	72.75±1.23	65.76±4.63	<0.001	47.48±2.30	22.17±3.14	<0.001
CPU wio (mean %)	3.88±1.06	0.70±0.16	<0.001	1.86±0.19	4.54±1.82	0.001
Bytes in (MB/sec)	1.15±0.09	2.12±0.42	<0.001	1.58±0.07	2.00±0.19	<0.001
Memory free (GB)	2.19±0.13	6.17±0.42	<0.001	0.91±0.07	0.70±0.03	<0.001

All times are presented as hr:min:sec and remaining metrics are shown as mean ± standard deviation.

*Calculated by paired *t*-test.

Although runtime variability was inevitable and present in both platforms when assembling each genome, GCE had a considerably greater variability with the larger human genome compared to EMR (coefficient of variation (COV)_EMR_ = 4.48% vs COV_GCE_ = 16.72%). We identified a single outlier in run time on GCE during the human genome analysis. This occurred due to the virtual cluster having a slower average network connection (1.55 MB/s compared to the average of the other GCE clusters of 2.02 MB/s) and a higher CPU WIO percentage than the average for the other GCE runs (9.56% versus 3.52%). The variation in cluster performance likely reflects an increase in network congestion amongst GCE servers.

Runtime predictably is an important issue in undedicated cloud computing. The existing workload of the cloud at the time of service usage is one of the main determinants of variability in runtime of undedicated services [12]. In our benchmarking, EMR was more consistent, though slower, in overall wall-clock time compared to GCE. This may suggest that GCE is more susceptible to server congestion than EMR; though service usage data is difficult to obtain.

## Discussion

Our findings confirm that cloud computing is an efficient and potentially cost-effective alternative for analysis of large genomic datasets. Cloud computing offers a dynamic, economical and versatile solution for large-scale computational analysis. There have been a number of recent advances in bioinformatic methods utilising cloud resources [4], [9], [13], and our results suggest that a standard genomic alignment is generally faster in GCE compared to EMR. The time differences identified could be attributed to the hardware used by the Google and Amazon for their cloud services. Amazon offers a 2.0 GHz Intel Xeon Sandy Bridge CPU, whilst Google uses a 2.6 GHz Intel Xeon Sandy Bridge CPU. This clock speed variability is considered the main contributing factor to the difference between the two undedicated platforms. It must also be noted that the resource requirements of Ganglia may have had a small impact on completion times [11].

There are a number of technical differences between GCE and EMR, which are important to consider when running standard bioinformatic pipelines. Running Crossbow on Amazon Web Services was simplified by an established support service, which provides an interface for establishing and running Hadoop clusters (Text S1). In contrast, there is currently no built-in support for GCE in Crossbow (Text S2). The current process to run a Crossbow job on GCE requires users to complete various steps such as installing and configuring the required software on each node in the cluster, transferring input data onto the Hadoop Distributed File System (HDFS), downloading results from the HDFS and terminating the cluster on completion. All of these steps are automatically performed by Crossbow on EMR. Python scripts offering similar functionality for GCE that Crossbow provides for EMR were created and are available (https://github.com/hewittlab/Crossbow-GCE-Hadoop).

While our findings confirm that cloud computing is an attractive alternative to the limitations imposed by the local environment, it is noteworthy that better performance metrics and lower cost were found with GCE compared to its established counterpart, Amazon’s EMR. Currently, a major limitation of these services remains at the initial transfer of large datasets onto the hosted cloud platform [14]. To circumvent this in the future, sequencing service providers are likely to directly deposit data to a designated cloud service provider, thereby eliminating the need for the user to double handle the data transfer [15]. Once this issue is resolved, it is foreseen that demand for these services is likely to increase considerably, given the low cost, broad flexibility and good customer support for cloud services [15]. The development of additional tools specific to genomic analysis in the cloud, which offer flexibility in choice of providers, is clearly required.

## Methods

### Datasets and Analytical Pipeline

**Figure 3:**
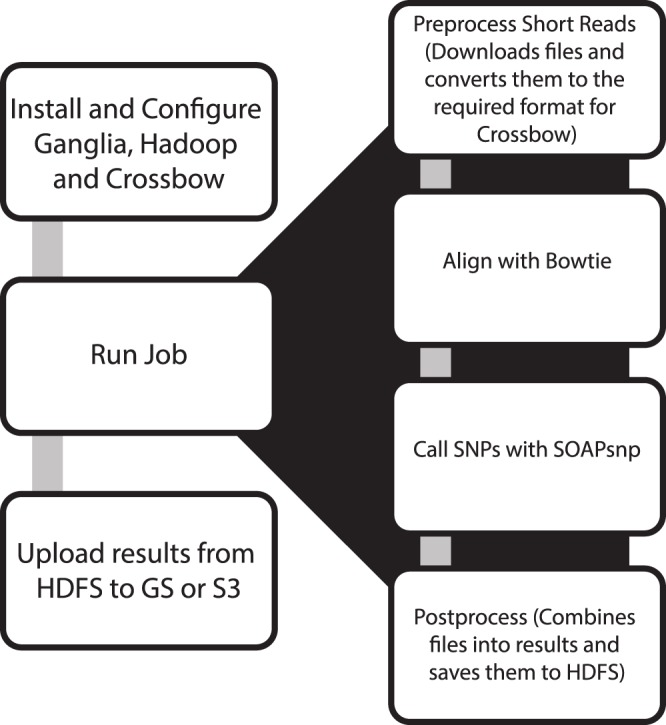
Analytical pipeline demarcating each step required to complete the Crossbow job in the cloud.

We benchmarked two platforms by a single job that completed read alignment and variant calling stages of next generation sequencing analysis simultaneously on two independent cloud platforms. To investigate the impact of data size on undedicated cluster performance, one small (*Escherichia coli* CC102 strain (3 GB SRA file; Accession: SRX003267) and one large (a Han Chinese male genome (142 GB Fastq files; Accession: ERA000005) publicly available genomic dataset was selected for analysis [8], [10]. For each job in this experiment, a parallel workflow was designed using Crossbow. This workflow included the following four steps: (1) Download and conversion of files; (2) Short read alignment with Bowtie; (3) SNP call with SOAPsnp; and (4) Combination of the results (Figure 3). Crossbow was the preferred genetic annotation tool in this experiment, as it has built in support for running via Amazon’s EMR and Hadoop clusters [16].

### Cluster construction and architecture

Instances were simultaneously established on Amazon’s EMR (http://aws.amazon.com/ec2/; Accessed 2014 Aug 7) and GCE (http://cloud.google.com/products/compute-engine.html; Accessed 2014 Aug 7). Undedicated clusters were optimized by selection of computational nodes as suggested for Crossbow [9]. Nodes with equivalent specifications were selected for each system (Table 2), these being c1.xlarge node in EMR and the closest specification node n1-highcpu-8 in GCE. For the *E.coli* genome, two nodes (one master and one slave) were used on each platform. On the other hand, for the human genome, the cluster was built with 40 nodes (one master and 39 slaves). As GCE did not provide any included storage for each instance, a 128 GB drive (the default storage quota provided by GCE) was added for each node. This was at the additional cost of $0.04/GB/Month or $0.000056/GB/Hour (Jan to June 2014).

**Table 2:** Specification of used computational nodes for each system.

	Virtual Cores	Memory (GB)	Included Storage (GB)	Price (USD/Hour)∧
**Amazon Elastic Compute Cloud (EC2)** **+ Elastic MapReduce (EMR) [c1.xlarge]**	8	7	4×420	$0.640
**Google Compute Engine [n1-highcpu-8]**	8	7.2	0#	$0.352

∧Date accessed: April to June 2014; prior to this period, pricing was $0.700 and $0.520 in Amazon and Google respectively.

# for each instance we added the minimum storage quota of 128 GB.

Each cluster was run using Apache Hadoop, an open-source implementation of the MapReduce algorithm [17]. MapReduce was used to organise distributed servers, manage the communication between servers and provide fault tolerance allowing tasks to be performed in parallel [18].

To explore the effect of network activity differences between the platforms, each job was run simultaneously; same day (including weekdays and weekends) and same time. Detailed description of the set up and scripts to run the jobs can be found in Text S1 and Text S2.

### Cluster Monitoring

**Figure 4:**
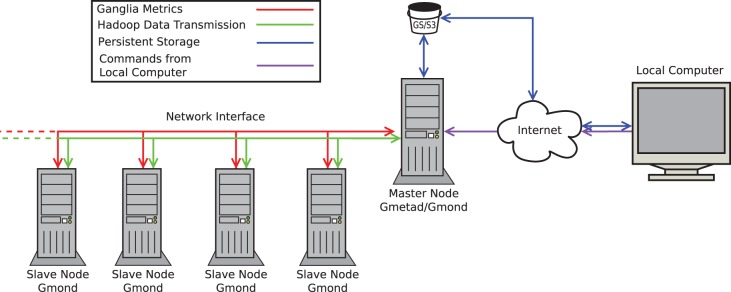
Directions and types of network transfers in our cloud-computing model. There are a variety of different network transfers between the nodes for each of the services in use in our model. Hadoop requires a bidirectional transmission of data between the master node and the slave nodes. This is required to coordinate the parallel processing of the cluster, and to allow for data transfer between nodes. Ganglia uses a unidirectional connection from the slave nodes to the master node to transfer the recorded metrics for storage and visualization. The persistent storage (provided by Amazon S3 (Simple Storage Service) or Google Storage, or an alternative method such as an FTP server) is accessed via the master node. The master node uses it to download input files for Crossbow, such as the manifest file and the reference Jar, and to use for persistent storage of the results of the Crossbow job as the instances destroy their storage on termination. Our local computer can also access the persistent storage via the Internet to allow access to upload the input files, or to download the results. The local computer needs to access the master node to initiate Crossbow. In EMR, this is replaced by a web interface and a JavaScript Object Notation Application Programming Interface (JSON API). In GCE, the user is required to remotely log in via Secure Shell (SSH) to commence the job.

In both EMR and GCE, multiple components of cloud infrastructure including CPU utilisation, memory usage and network speeds were monitored and recorded for each node using Ganglia. The default setting of Ganglia for distributing incoming requests is multicast mode; however, since EMR and GCE environments do not currently support multicast Ganglia, it was configured in unicast mode (Figure 4). The metric output files constructed in.rrd format were converted into.csv format with a Perl script (Text S3). For comparison between performance and costs between platforms, the Student t-test was undertaken using the statistical software R (R Foundation for Statistical Computing version 3.0.2; http://www.r-project.org/). In the analysis, cost of each run was calculated using current pricing (June 10^th^ 2014); however, all *E.coli* runs and one human genome run were performed prior to a recent decrease in price on both platforms. The COV for runtime variability was calculated as the ratio of the standard deviation to the mean time (mins) for each system.

## Supporting Information

Text S1 **Uploading data and setting up an Amazon Web Services Elastic MapReduce (EMR) cluster.**
(DOCX)

Text S2 **Scripts for configuration and running jobs on Google Compute Engine (GCE).**
(DOCX)

Text S3 **Transformation of metric outputs from. RRD to. CSV format.**
(DOCX)
