# Supplementary material for: Benchmarking Undedicated Cloud Computing Providers for Analysis of Genomic Datasets
Source: PLoS One. 2014 Sep 23;9(9):e108490. doi: 10.1371/journal.pone.0108490 (PMC4172764; doi:10.1371/journal.pone.0108490)
Supplement: Text S1 — Uploading data and setting up an Amazon Web Services Elastic MapReduce (EMR) cluster. (DOCX) [file pone.0108490.s001.docx]

**Supporting Information 1: Uploading data and setting up an Amazon Web Services Elastic MapReduce (EMR) cluster.**

Crossbow on EMR supports importing data referenced from the manifest file from HTTP, FTP, HDFS, and S3. We uploaded the input data (available from: <ftp://public.genomics.org.cn/BGI/yanhuang/Rawdata2>) into a S3 bucket in order to have the maximum potential download speed and ensure that the cluster was not sitting idle while the inputs were downloaded from a remote FTP server. The process used to upload the files from the remote FTP to our S3 bucket was as follows:

1. Create a micro instance on EC2, ensuring the instance has enough storage space for the whole dataset.
2. SSH into the instance using the given hostname,
3. (Optional) Install the screen package, and create a screen session. This allows you to close the SSH connection and allow the processes to continue. This is done via:
   1. sudo apt-get install screen
   2. Note: a screen session can be reconnected by:

screen –r

1. Download the data from the remote FTP server:
   1. wget ‑r ftp://public.genomics.org.cn/BGI/yanhuang/Rawdata2
2. Upload the data to the S3 bucket

s3cmd put --recursive /path/to/data/ s3://<your‑bucket>/path/to/save/data

The time taken to download the 142GB human genome data from the FTP server was 10:35:49 and the time taken to upload to the S3 bucket was 2:41:30, giving a total transfer time of 13:17:19.

The Ganglia install scripts provided by Amazon were using an out-of-date version of the software, which did not allow the metric data to be exported. As such these install scripts were updated to use the current version of Ganglia and Ganglia-web (3.6.0 and 3.5.10 respectively) and reinstalled.  Ganglia was configured to use unicast mode as EMR prevented use of multicast.

Crossbow provided a faulty file in their S3 bucket that consistently caused the final step on EMR to fail. The file was hosted on our development bucket and Crossbow was modified so it was pointing to the S3 bucket. The link in the source code of Crossbow was altered and should now be corrected in the official Crossbow bucket.
