# Supplementary material for: Benchmarking Undedicated Cloud Computing Providers for Analysis of Genomic Datasets
Source: PLoS One. 2014 Sep 23;9(9):e108490. doi: 10.1371/journal.pone.0108490 (PMC4172764; doi:10.1371/journal.pone.0108490)
Supplement: Text S2 — Scripts for configuration and running jobs on Google Compute Engine (GCE). (DOCX) [file pone.0108490.s002.docx]

**Supporting Information 2: Scripts for configuration and running jobs on Google Compute Engine (GCE).**

Crossbow provides an option that sets up and configures an EMR cluster with the required software. Although no such support is available for GCE, Crossbow supports use of a Hadoop Cluster that can be implemented in GCE. Google have released scripts for creating a Hadoop cluster on the GCE services (available from: <https://github.com/GoogleCloudPlatform/solutions-google-compute-engine-cluster-for-hadoop>). However, these scripts needed to be modified as we required some additional software to be installed on each node of the cluster. These modifications can be found at <https://github.com/hewittlab/Crossbow-GCE-Hadoop> . The modifications perform the following additional steps when setting up the cluster:

1. Create Ganglia configuration files and Hadoop configuration files specifically for the hostnames and IP addresses assigned to each of the nodes in the cluster.
2. Upload the configuration files to the Google Storage Bucket.
3. Install the required software on each of the nodes in the cluster, and place the configuration files from step 1 into their appropriate locations.

To run Crossbow on GCE, we initially uploaded the input reference Jar and the input manifest file to the HDFS. Given that these files were to be used for multiple runs and to avoid re-upload these files multiple times this was performed via the Google Storage Bucket. Once completed, the cluster was started. The input files were then downloaded from the bucket, onto the master node and then into the HDFS. Crossbow for Hadoop was then run with the appropriate parameters for the current cluster and input files, with the output files being copied from the HDFS to the bucket on completion. Each cluster was terminated upon completion of the workflow.

To reduce the repetition of steps required to set up the GCE cluster for each experimental run, an additional script, run_crossbow.py was created. While this script simplified the process to a level that was comparable to running Crossbow for EMR on the command line, it also raised several other issues. For example, access permission to the RRD directory on each node was made available to all user as required by Ganglia using ‘chown’ command.

Hadoop is designed to manage multiple failures and prevent a job from completely failing within a cluster. The default configuration of Hadoop stops the cluster immediately in case of a single failure. To prevent random task failures stopping the GCE cluster, we increased the threshold number of failed tasks allowable within the cluster.

When running Hadoop on GCE, a storage disk space had to be allocated to the instance at the time of its creation. Moreover, when creating a disk based off a Debian image, the available disk space was limited to 10GB regardless of the specified disk size. Linux partitions were edited to utilize the full size of the disk. A snapshot of the storage disk was created in order to avoid repartitioning of the disk during node creation. These modifications were updated further in the release version to reduce the setup time.

By default, Google only allows access to a limited number of resources per project. Since use of large cluster recommended in Crossbow runs, we applied for access to additional number of cores, IP addresses and total disk space in the region of our choice through an online form provided on Google Developers Console.

On January 9^th^ 2014, Google depreciated the v1beta15 application programming interface (API) and disk used for temporary storage was replaced with a persistent disk approach. This change caused problems in our initial cluster setup. Therefore, setup scripts were altered in order to generate a disk for each node prior its creation and to delete these disks upon termination. To point our code to the new API, the cluster creation scripts were modified based on a revised version of the Google’s code. However, later on we rewrote our changes into the updated Google scripts which are now available in our repository.

Finally, it is important to configure the number of Crossbow tasks appropriately to ensure complete node usage. The following arguments should be used with Crossbow on GCE: --cpus (number of cores per instance); and --instances (number of instances). It is important to note that these arguments are applicable to Hadoop in a non-EMR environment.
