# Supplementary material for: Benchmarking Undedicated Cloud Computing Providers for Analysis of Genomic Datasets
Source: PLoS One. 2014 Sep 23;9(9):e108490. doi: 10.1371/journal.pone.0108490 (PMC4172764; doi:10.1371/journal.pone.0108490)
Supplement: Text S3 — Transformation of metric outputs from. RRD to. CSV format. (DOCX) [file pone.0108490.s003.docx]

**Supporting Information 3: Transformation of metric outputs from .RRD to .CSV format.**

The program rrd2csv.pl was used to convert RRDs to CSVs. It is available from <https://code.google.com/p/rrd2csv/>. Its usage is:

perl rrd2csv.pl -s “17/12/2013 12:00” -e “17/12/2013 13:00” file.rrd

To store the output as a csv, add “> file.csv” to the end (i.e perl rrd2csv.pl -s “17/12/2013 12:00” -e “17/12/2013 13:00” file.rrd > file.csv )

The -s and -e flags are representing the times that one adds the csv, where -s is the start time and -e is the end time. So in the example above, we are exporting from today at 12, to today at 1pm. These times should be when the job ran.
